# Evaluation of a Surgical Navigation System for Localization and Excision of Nonpalpable Lesions in Breast and Axillary Surgery

**DOI:** 10.1155/2023/9993852

**Published:** 2023-12-23

**Authors:** Kjirsten A. Carlson, Cristina Checka, Kelly K. Hunt, Jennifer Jung, Christian Bridges, Puneet Singh, Ana Refinetti, Tanya Moseley, Frances Perez, Cody Mayo, Nina Tamirisa

**Affiliations:** ^1^Department of Breast Surgical Oncology, The University of Texas MD Anderson Cancer Center, Houston, TX 77030, USA; ^2^Department of Breast Diagnostic Imaging, The University of Texas MD Anderson Cancer Center, Houston, TX 77030, USA

## Abstract

**Introduction:**

Elucent Medical has introduced a novel EnVisio™ Surgical Navigation system which uses SmartClips™ that generate a unique electromagnetic signal triangulated in 3 dimensions for real-time navigation. The purpose of this study was to evaluate the efficacy and feasibility of the EnVisio Surgical Navigation system in localizing and excising nonpalpable lesions in breast and axillary surgery.

**Methods:**

This pilot study prospectively examined patients undergoing breast and nodal localization using the EnVisio Surgical Navigation system. SmartClips were placed by designated radiologists using ultrasound (US) or mammographic (MMG) guidance. The technical evaluation focused on successful deployment and subsequent excision of all localized lesions including SmartClips and biopsy clips.

**Results:**

Eleven patients underwent localization using 27 SmartClips which included bracketed multifocal disease (*n* = 4) and clipped lymph node (*n* = 1). The bracketed cases were each localized with 2 SmartClips. Mammography and ultrasound were used (*n* = 8 and *n* = 19, respectively) to place the SmartClips. All 27 devices were successfully deployed within 5 mm of the targeted lesion or biopsy clip. All SmartClip devices were identified and retrieved intraoperatively. No patients required a second operation for margin excision.

**Conclusion:**

In a limited sample, the EnVisio Surgical Navigation system was a reliable technology for the localization of breast and axillary lesions planned for surgical excision. Further comparative studies are required to evaluate its efficacy in relation to the other existing localization modalities.

## 1. Introduction

Breast conserving therapy (BCT) has been part of the standard of care in treating breast cancer for over 25 years [1]. Survival and recurrence outcomes are comparable to mastectomy, with recent retrospective data suggesting a survival advantage among BCT patients in some cases [2]. Prior studies have shown improved patient satisfaction with BCT compared to mastectomy with reconstruction [3]. Eligibility for BCT has expanded to patients with multifocal/multicentric disease, including patients receiving neoadjuvant chemotherapy, as a safe and effective strategy in conjunction with oncoplastic reconstruction [4, 5].

Image-guided localization has allowed for more precise resection of nonpalpable disease with improved oncologic and aesthetic outcomes in patients undergoing BCT. Wire localization (WL) was initially established as the standard for preoperative localization of breast lesions using mammographic or ultrasound (US) guidance. However, over time, WL has been associated with multiple disadvantages including coordination of scheduling between radiology and surgery, patient dissatisfaction with the wire protruding from their breast prior to excision, and the possibility of wire dislodgement before it is removed. Additionally, the trajectory of the wire placement can interfere with the surgeon's ability to excise the lesion. Thus, new technologies have been developed with the goal of accurately localizing breast lesions while improving both the surgeon and patient experience [6].

Examples of these new technologies include the use of radioactive seeds (RS), magnetic seeds (MS), and SAVI scout radar (SSR). One important advantage of the aforementioned devices is the decoupling of radiology and OR schedules since patients can have localizers placed on a different day from the surgical procedure. The SSR can even be placed at the time of diagnosis and remain in situ throughout the neoadjuvant therapy. Improvements have been observed in the overall patient and physician experience with these new devices when compared to WL, while maintaining accuracy of localization [7–10].

The RS uses an I-125 seed for localization of the breast lesion and a gamma probe for intraoperative detection. The MS uses a stainless steel marker detected intraoperatively with a probe which generates a magnetic field to identify the magnetized seed. The SSR uses radiofrequency or electromagnetic technology to detect reflectors or tags implanted within the breast or nodal lesion [9, 11]. While these devices streamline the localization process, each has advantages over the others. The radar signal of the SSR can be weakened or disrupted if exposed to certain operating room lights or deactivated when in close proximity to electrocautery [12]. In a retrospective study from our institution, the MS overcame regulatory constraints and mandatory isotope tracking associated with the RS without compromising surgical outcomes [13]. However, the MS requires the use of nonmetallic instruments when using the detection probe to avoid signal interference which may in turn limit operative exposure [9].

**Figure 1:**
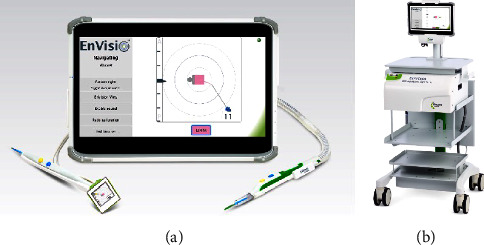
EnVisio Surgical Navigation system; (a) wireless display screen showing intraoperative navigation with two options of transducers and (b) system console with detachable wireless screen.

Elucent Medical has introduced a novel EnVisio™ Surgical Navigation system which uses SmartClips™ that generate a distinctive electromagnetic signature detected by a transducer placed on the surgeon's electrocautery device and then displayed on the navigation system's wireless screen. The SmartClips are approved by the Food and Drug Administration (FDA) to be placed at any time prior to the surgical procedure and do not require removal for regulatory purposes. The average selling price for the individual SmartClip preloaded in an introducer is $450. At the time of surgery, an electromagnetic pad is placed under the patient on the operative table which provides a unique signal for each SmartClip. The location of the SmartClip is then triangulated in the *x*-, *y*-, and *z*-axes using real-time wireless navigation detected by the transducer (Figures 1(a) and 1(b)). The transducer sends this information to the navigation system's screen which displays the distance from the smart clip to the tip of the electrocautery device within millimeters in all 3 dimensions. Standard surgical instruments do not interfere with these signals, allowing the surgeon to use any instruments for optimal exposure [12]. There are no studies to date evaluating the accuracy of the EnVisio Surgical Navigation system in the targeted excision of breast and nodal lesions. The purpose of this pilot study was to evaluate the efficacy and feasibility of the EnVisio Surgical Navigation system in localizing and excising targeted breast lesions and axillary lymph nodes within our hospital system.

## 2. Materials and Methods

**Figure 2:**
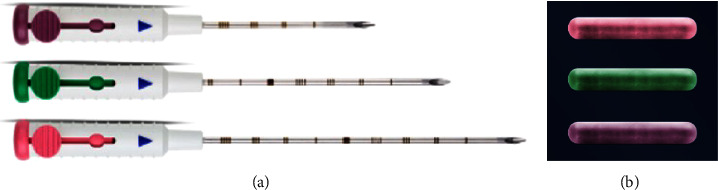
(a) 15-gauge SmartClip deployment device; (b) SmartClip (1.4 mm × 8 mm) in various colors for ease of bracketing or localizing multiple lesions.

**Figure 3:**
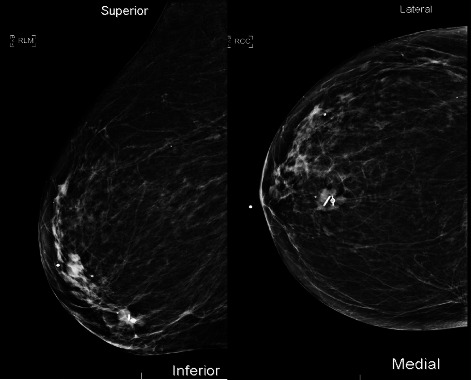
Postprocedure mammography showing accurate SmartClip localization of biopsy clip.

We prospectively examined patients undergoing preoperative localization of breast and axillary lesions using the EnVisio Surgical Navigation system at the University of Texas MD Anderson Cancer Center (MDACC) (*n* = 22) from August 2021 through February 2022. Institutional Review Board approval was obtained and Elucent Medical provided all products at no charge during the clinical cases used in this analysis. Patients requiring preoperative localization of nonpalpable breast and/or axillary lesions were identified and consented using standard institutional protocols. Localization was performed prior to the date of surgery based on surgeon and patient preference. Designated radiologists (*n* = 10) underwent training on the deployment device by EnVisio personnel. The 15-gauge SmartClip deployment device was used to place the localizing SmartClip at the targeted lesion using either ultrasound (US) or mammographic (MMG) guidance (Figure 2(a)). The 1.4 mm × 8 mm SmartClip is available in multiple colors each with its own individualized electromagnetic signature for bracketing of a single lesion or localization of multiple lesions within the breast (Figure 2(b)). Once localization was completed, a postprocedure MMG was obtained to evaluate accuracy of localization which was measured by the distance from SmartClip to biopsy clip in both the cranio-caudal (CC) and medial-lateral (ML) views (Figure 3).

In preparation for surgery, the EnVisio system electromagnetic pad was positioned on a compatible operating table underneath the patient and the transducer was placed on the surgeon's electrocautery device. The pad generated electromagnetic waves to detect and track the SmartClip location relative to the transducer which was attached to the electrocautery device. This signal was then triangulated in the 3-dimensional *x*-, *y*-, and *z*-axes using real-time wireless navigation and displayed on the navigation screen (Figure 1(a)). The distance in millimeters between the tip of the electrocautery device and each individual SmartClip, designated by color, was displayed on the navigation screen.

**Figure 4:**
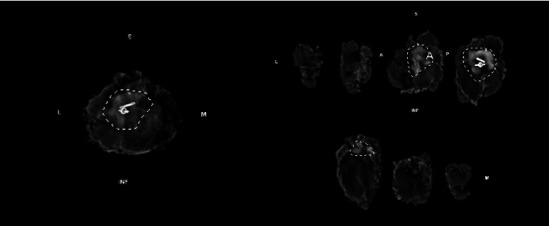
Breast specimen radiograph showing intraoperative radiologic evaluation of margins.

A total of eight surgeons participated in the study. Surgical excision was performed in standard fashion per the surgeon's preferred technique. The breast excised specimen was then oriented per institutional protocol and the transducer was used to confirm the presence of the SmartClip within the specimen. If the SmartClip was not identified within the primary specimen, the surgical cavity was reexamined with the transducer and an additional margin was obtained to excise the SmartClip. The primary specimen was sent for both radiologic and pathologic evaluation to ensure removal of both the SmartClips, the target lesion and the biopsy clip (Figure 4). It is our institutional practice to have real-time intraoperative evaluation of specimen margins. If the radiologist or pathologist observed abnormality close to a margin on specimen radiograph or on gross examination, an additional margin was excised in that area.

Final pathologic results were reviewed and additional data were obtained regarding patient and tumor characteristics. The primary endpoint of the study was rate of successful excision of the targeted lesion. Secondary endpoints included number of additional margins obtained based on preliminary assessment of the intact specimen, final tumor margins, accuracy of localization, complications or safety related concerns, and rates of reexcision. Successful excision of the targeted lesion was defined as removal of both the biopsy clip and SmartClip at the index surgery. Accurate localization of the lesion was defined as SmartClip placement within 5 mm range of the biopsy clip/targeted lesion. Descriptive statistics (proportions, frequencies, means, and medians) were generated.

## 3. Results

### 3.1. Patient and Tumor Characteristics

A total of 22 patients underwent localization of breast or axillary lesions using 27 SmartClips; these included bracketed lesions (*n* = 4) and clipped lymph nodes (*n* = 1). The bracketed cases were each localized using 2 SmartClips. Patient and tumor characteristics are shown in Table 1. Patients had a mean BMI of 26.9 (22–44). Two patients (9.1%) had a history of previous breast surgery in the ipsilateral breast and no patient had a history of prior ipsilateral breast irradiation (XRT). Roughly half of the patients received neoadjuvant chemotherapy (*n* = 9, 52.9%). Median tumor size was 1.5 cm (range 0.4–3.6 cm) and two patients (9.1%) had multifocal disease. Preoperative histopathology (*n* = 22) consisted of atypia (*n* = 1), ductal carcinoma in situ (DCIS) (*n* = 4), invasive ductal carcinoma (IDC) (*n* = 12), invasive lobular carcinoma (ILC) (*n* = 2), mixed IDC/ILC (*n* = 1), IDC with DCIS (*n* = 1), and invasive mucinous carcinoma (*n* = 1). Of the malignant lesions, presenting stages ranged from stage 0 (Tis) to stage IIIB.

### 3.2. Localization

US was used for localization in the majority of cases (*n* = 19). No biopsy clips had migrated from the targeted lesion when the patient was taken for radiologic localization. All SmartClips were accurately placed within 5 mm of the targeted lesion or biopsy clip (*n* = 27) and there were no reported complications or adverse events during or after the localization procedure.

### 3.3. Surgical Excision (Table 2)

All SmartClips were successfully retrieved during the surgical procedure. The SmartClip was removed with the primary specimen in *n* = 22 (81.5%) of cases and sent as a separate specimen in *n* = 4 (14.8%) cases. Retrieval of the SmartClip as an additional margin occurred in one patient who had a bracketed clinical stage T2 ILC. This additional margin contained ILC and served as the final margin which was negative. The median operative time from incision to removal of the all SmartClips was 18.3 minutes (10.5–49.4 minutes). The mean volume of the breast tissue resected was 35.4 cm^3^. The median tumor margin was 3 mm (3–5 mm) for patients with DCIS and 5 mm for patients with invasive disease (2–8 mm). No patients required reexcision for positive margins. Difficulty locating and excising the SmartClip occurred in one patient with localized nodal disease which required lateral decubitus positioning intraoperatively. It was noted that this positioning increased the distance between the SmartClip and the underlying electromagnetic pad making nodal localization difficult to reproduce. Patients were in the supine position for all other cases (*n* = 21) and the SmartClips remained within the field produced by the electromagnetic pad.

## 4. Discussion

The EnVisio Surgical Navigation system was found to be a reliable technology for the localization and excision of nonpalpable breast and axillary nodal lesions. In this small cohort of patients, the retrieval of the SmartClip required excision of an additional margin in 1 case, however, all clips were removed at the index operation. There were no positive or close margins on final pathology. No patients required a reoperation for reexcision of margins or retrieval of the SmartClip. To date, this is the first study to evaluate the feasibility of the EnVisio Surgical Navigation system in localizing targeted breast and axillary lesions.

In our study, all localized biopsy clips and lesions of interest were successfully excised with intraoperative radiographs confirming removal of all biopsy clips within the primary specimen. This is also a function of accurate localization by the radiologists prior to surgery as all SmartClips were placed within 5 mm of the targeted lesion or biopsy clip, and no biopsy clips were reported to have migrated on postprocedure mammography. Furthermore, there were no cases of close or positive margins on final pathology with use of the EnVisio Surgical Navigation system and no reoperations for reexcision of margins were required. In comparison, SSR has been reported to have a 7.4% positive margin rate [10]. In a 2021 multicenter randomized clinical trial in Australia, the reexcision rate with RS was 13.9% which was significantly lower than that of wire localization (18.9%) [11]. Mitigating risks associated with additional surgery, lowering cost of care, and alleviating patient stress associated with the need for additional surgery drive continued efforts to reduce rates of reexcision. While the sample size of our study is too small for meaningful statistical analysis, the results are favorable and warrant further study with an increased number of patients.

The EnVisio Surgical Navigation system was noted to be useful in cases where lesions were bracketed or multiple lesions were excised in the same breast. In prior studies, bracketing of breast lesions using multiple nonwire localizing devices allowed for BCT in patients who would have otherwise required mastectomy with acceptable oncologic and aesthetic outcomes [14, 15]. However, while the MS, SSR, and RS only detect one common signal, the SmartClip allows the surgeon to differentiate localizing signals from each SmartClip. When the EnVisio transducer was within close proximity of a specific SmartClip, the color of the clip was displayed on the navigation screen along with the clip's distance from the tip of the electrocautery device in 3 dimensions. The surgeon was able to toggle between the SmartClips and guide excision using the designated colors. The ability to distinguish between signals is particularly useful when multiple localizers are positioned in the anterior-posterior dimension of the targeted lesion or when located within close proximity to one another.

With respect to use of the EnVisio transducer, one advantage is the ability to use the standard surgical instruments without the need for additional probes to detect and localize the SmartClip. The EnVisio transducer, which is single use, attaches directly to the electrocautery device which eliminates the need for probes that typically require sterilization between use, continued maintenance, and high cost of repairs for inadvertent damage or malfunction. Additionally, the surgeon is able to view navigation in real-time using the portable touch screen display which can be placed on an IV pole for optimal viewing during surgery.

In terms of complications and contraindications of the device, the system does have limitations. In 5 instances, the SmartClip was removed separately from the primary surgical specimen. Four of these were sent as separate gross specimen due to displacement from the breast parenchyma during manipulation of the surrounding tissue. SmartClips have a smooth exterior coating which decreases its ability to anchor into the surrounding tissue. This exterior coating is currently undergoing review for improvement based on previously reported feedback from surgeons. In the one instance of the SmartClip being removed as an additional margin, the patient presented with a 3.0 cm ILC that underwent neoadjuvant therapy. This lesion was bracketed using two SmartClips, and at the time of excision, only one SmartClip was identified in the primary specimen while the other was removed as the additional margin. This additional margin contained ILC; however, no tumor was present on final inked margin. Compared to WL and SSR, the SmartClip appears to have a lower rate of retrieving both the biopsy clip and localization device within the same primary specimen than the aforementioned localizing techniques (80.7% SmartClip versus 96.2% WL and 94.3% SSR) [6].

Another limitation is that patients must be placed in the supine position for the duration of surgery in order for the SmartClip to stay within the confines of the electromagnetic pad on the operating table. A patient's body habitus or alternative positioning could preclude the localizing signal from being detected if positioned too far away from the pad. Thus, the aforementioned difficulties encountered with left lateral decubitus positioning may also prohibit certain reconstruction techniques (i.e., lateral wall perforator flap reconstruction).

One contraindication for use of the navigation system is presence of an active cardiac device that could interfere with the signal from the electromagnetic pad. However, a magnet placed on the cardiac device to deactivate it during the operation allows for the use of the system. Patients with the SmartClip can be scanned safely with magnetic resonance imaging (MRI); however, the image artifact caused by the device extends approximately 20 mm and the SmartClip cannot be placed under MRI guidance. Regarding implantable devices, Elucent Medical has continued to test routine medical implants for interference with the navigation system, and to date, there has not been restrictions to its performance. Additionally, the electromagnetic pad that is placed under the patient precludes the use of certain intraoperative equipment that may interfere with the signal such as extra padding often used for anticipation of prolonged cases. Furthermore, the electromagnetic pad interferes with imaging obtained from intraoperative fluoroscopy which cannot be used for detection of clips missing from the resected specimen. Finally, the operating room table to be used during surgery must also be reviewed with the Elucent representative prior to utilization of SmartClips. While majority of operating room tables are compatible with the device, a select few may interfere with the navigational coordinates displayed during the operation.

Our study is limited given its single-institutional nature and small study population. While this was a feasibility study of the device and the initial results are favorable, further study with increased numbers of patients is required to confirm these preliminary findings. With respect to margin assessment and need for reexcision, it must be recognized that the MD Anderson Cancer Center has an institutional protocol for intraoperative radiologic and pathologic margin assessment of the breast specimen. This practice thus interferes with the generalizability of this study's reexcision rate to other institutions.

## 5. Conclusion

In conclusion, the EnVisio Surgical Navigation system is a safe and reliable emerging technology for the localization of breast and axillary lesions planned for surgical excision. This device may be particularly useful for bracketed lesions given the SmartClip's unique localization information. Larger studies are warranted that evaluate the efficacy of this system within a diverse patient population and broad variety of clinical settings. Future studies should also assess the feasibility of adopting this new technology within various practice models.

## Figures and Tables

**Table 1:** Patient and tumor characteristics of EnVisio Surgical Navigation system localized cohort (*n* = 22).

	*N* = 22
Age (years)	62 (42–81)
BMI	26.9 (22–44)
History of previous ipsilateral breast surgery	2 (9.1%)
Received neoadjuvant chemotherapy^*∗*^	9 (52.9%)
Median tumor size (cm)	1.4 5 (0.4–3.6)
Multifocal	2 (9.1%)
Preoperative histopathology	
Atypia	1 (4.5%)
DCIS	4 (18.2%)
IDC	12 (54.5%)
ILC	2 (9.1%)
Mixed IDC/ILC	1 (4.5%)
IDC with DCIS	1 (4.5%)
IMC	1 (4.5%)

^*∗*^Neoadjuvant chemotherapy is only offered to patients with invasive tumors and the denominator for this particular variable was *n* = 17. DCIS, ductal carcinoma in situ; IDC, invasive ductal carcinoma; ILC, invasive lobular carcinoma; IMC, invasive mammary carcinoma.

**Table 2:** Surgical and pathologic details of surgical excision using EnVisio Surgical Navigation system.

	*N* = 27
Devices excised within primary specimen	22 (81.5%)
SmartClip sent as separate gross specimen	4 (14.8%)
SmartClip in the same specimen as biopsy clip	22 (81.5%)
Retrieval of SmartClip as additional margin	1 (3.7%)
Median tumor margin: DCIS	3.0 mm (3–5)
Median tumor margin: invasive	5.0 mm (2–8)
Reexcision performed	0

DCIS, ductal carcinoma in situ; mm, millimeter.

## Data Availability

The data for this study can be found in a protected database at The University of Texas MD Anderson Cancer Center due to patient privacy.
